# Chemsex behaviours, sexual response and sexual health

**DOI:** 10.1192/j.eurpsy.2023.1340

**Published:** 2023-07-19

**Authors:** J. Curto Ramos, I. Azqueta, M. T. Heredia, R. Molina Prado, I. De Ema López

**Affiliations:** 1 Department of Psychiatry, Clinical Psychology and Mental Health, La Paz University Hospital, Madrid, Spain, Hospital la Paz; 2 NGO Apoyo Positivo, Madrid; 3 NGO Apoyo Positivo, Málaga; 4 CAD ARGANZUELA. Instituto de Adicciones Madrid Salud; 5 CAD TETUÁN. Instituto de Adicciones Madrid Salud, Madrid, Spain

## Abstract

**Introduction:**

The intentional use of drugs before or during sexual intercourse (chemsex) is a phenomenon of special importance in the MSM (men who have sex with men) population due to its impact on mental, physical and sexual health. Sexual health issues related to chemsex practice have been described such as difficulties in achieving sober sex, erectile dysfunction or problems with sexual desire.

**Objectives:**

The objective of this study was to understand the impact of chemsex on sexual health and sexual response by the participantes of a sexual health program for chemsex users in two Substance Use Disorder Clinics in Madrid.

**Methods:**

Qualitative research approach. We analyze an anonymous survey with chemsex users with open answer questions about the impact of chemsex practice on sexual response and sexual health. Data analysis was based on thematic analysis of content.

**Results:**

Several differences were identifed between chemsex and sober sex. In sober sex it can take longer to feel aroused, sexual desire is more context-dependent and more easyly controled. They connect easily with other people needs when they had sober sex. They described difficulties with consent with some sexual practices when they were on drugs. Shame and guilt was associated with chemsex. They describe more arousal, more independent of the erotic context, longer sexual intercourse and delayed ejaculation when they had sex under the influence of drugs.

**Conclusions:**

Chemsex is a phenomenon that needs a multidisciplinary approach and mental and sexual health must be taken into account including sexological perspective. Interventions that provide sexual counselling and sexual therapy for chemsex users must be developped.

**Disclosure of Interest:**

None Declared

